# Tamoxifen reverses epithelial–mesenchymal transition by demethylating miR-200c in triple-negative breast cancer cells

**DOI:** 10.1186/s12885-017-3457-4

**Published:** 2017-07-19

**Authors:** Qian Wang, Yu Cheng, Yan Wang, Yibo Fan, Ce Li, Ye Zhang, Yiding Wang, Qian Dong, Yanju Ma, Yue-e Teng, Xiujuan Qu, Yunpeng Liu

**Affiliations:** 1 Department of Medical Oncology, The First Hospital of China Medical University, Shenyang, Liaoning 110001 People’s Republic of China; 2 Department of Medical Oncology, Liaoning Cancer Hospital and Institute, Cancer Hospital of China Medical University, Shenyang, Liaoning 110042 People’s Republic of China; 3 Department of Oncology, Shengjing Hospital of China Medical University, Shenyang, Liaoning 110004 People’s Republic of China; 4 Department of Urology, Liaoning Cancer Hospital and Institute, Cancer Hospital of China Medical University, Shenyang, Liaoning 110042 People’s Republic of China; 5 Key Laboratory of Anticancer Drugs and Biotherapy of Liaoning Province, the First Hospital of China Medical University, NO. 155, North Nanjing Street, Heping District, Shenyang, 110001 China

**Keywords:** Tamoxifen, Epithelial to mesenchymal transition, Triple negative breast cancer, Demethylation, MicroRNA

## Abstract

**Background:**

Although the efficacy of tamoxifen (TAM) for breast cancer has been attributed to inducing cell cycle arrest and apoptosis by inhibiting estrogen receptor (ER) signaling, recent evidence indicates that TAM also possesses ER-independent antitumor activity through an unclear mechanism. The present study investigated the anti-tumor mechanism of TAM on mesenchymal triple-negative breast cancer (TNBC).

**Methods:**

The inhibitory effect of TAM on tumor migration and metastasis was analyzed by transwell chamber in vitro and by murine xenograft model in vivo. The promoter sequence of miR-200c was predicted by an online CpG island predictor. Relative expression of miR-200c was measured by quantitative real-time PCR.

**Results:**

After treatment with TAM, mesenchymal TNBC cells (MCF-7/ADR and MDA-MB-231) morphologically changed from mesenchymal to epithelial types. Meanwhile, cell migration ability was also significantly decreased in ER-positive breast cancer cells after exposure to TAM. Consistent with these in-vitro results, TAM significantly suppressed lung metastasis rate of mesenchymal TNBC cells in murine xenograft tumors. miRNA array analysis of two types of breast cancer cells showed that miR-200c expression was inhibited in mesenchymal TNBC cells, but increased after TAM treatment due to demethylation of miR-200c promoters.

**Conclusions:**

Our results indicate that TAM inhibits cell migration and enhances chemosensitivity of mesenchymal TNBC cells by reversing their EMT-like property; and that this EMT-reversal effect results from upregulation of miR-200c through demethylating its promoter. To our knowledge, this is the first explanation of a non-ER-related mechanism for the effect of TAM on mesenchymal TNBC cells.

**Electronic supplementary material:**

The online version of this article (doi:10.1186/s12885-017-3457-4) contains supplementary material, which is available to authorized users.

## Background

Wider use of personalized targeted and endocrine-based therapies has significantly improved outcomes of *Her2* amplificated and hormonal receptor (HR)-positive (HR ^+^) breast cancer subtypes. However, triple negative breast cancer (TNBC) is still associated with high recurrence and short survival. Because TNBC lacks biological targets, it is mainly treated with chemotherapy. However, chemosensitivity in TNBC is limited and urgently needed to be improved.

Epithelial-to-mesenchymal transition (EMT) is a part of tumor metastasis, which is characterized by decreased epithelial marker E-cadherin and increased mesenchymal marker vimentin, is a subprocess of both tumor metastasis and drug resistance development [1]. Increased vimentin expression has been related to taxane residues in ovary and breast cancer cells which shows drug resistance property [2, 3]. EMT can also induce anthracycline resistance in cancer cells [4]. As most of TNBC cells have a mesenchymal phenotype, EMT might be the major cause of TNBC multidrug resistance. Determining the EMT regulatory pathways and reversing the EMT process might thus improve TNBC chemosensitivity.

Tamoxifen (TAM) is commonly used in HR^+^ breast cancer with more than 50% effectiveness [5], whereas the effectiveness of TAM in estrogen receptor negative (ER^−^) breast cancer is reportedly about 7% [6]. Recently, TAM was shown to exert an antitumor effect in ER^−^ cancers, including gastric cancer, colon cancer and cholangiocarcinoma [7–9]. Although the antitumor mechanism of TAM is considered to be competition with estrogen to block ER transcription, researchers have found a non-estrogen-dependent mechanism of TAM in ER^−^ cancers by activating the apoptosis pathway to induce apoptosis. However, the apoptotic mechanism does not explain TAM activity in all types of ER^−^ cancers. Other non-estrogen-dependent mechanisms for TAM has been suggested, such as the mediation of protein kinase C (PKC), transforming growth factor-β (TGF-β), oncogene *c-myc* and mitogen-activated protein kinase (MAPK) [10, 11]. The relationship between EMT and TAM has been established in ER^+^ breast and endometrial cancers [12]. Most researchers indicated that long-time use of TAM might induce TAM resistance, which could induce EMT in ER^+^ cancers [1]. However, the relationship between TAM and EMT in ER^−^ cancers, especially TNBC, is unclear.

In this report, our result revealed that TAM could reverse EMT characteristics in mesenchymal TNBC cells, but not epithelial breast cancer cells. Further study indicated that reversing EMT enhanced chemosensitivity. These results imply a possible clinical indication for TAM in TNBC.

## Methods

### Cell lines and reagents

Human breast cancer cell lines MCF-7 (TCHu74) and MDA-MB-231 (TCHu227) were obtained from the Cell Bank of the Chinese Academy of Sciences (China). MCF-7/ADR cells derived from MCF-7 and cultured with 1 μg/mL adriamycin for at least 1 year and possessed adriamycin-resistance [13]. Human breast cancer cell lines MCF-7 and MCF-7/ADR cells were cultured in RPMI 1640 medium (GIBCO, Grand Island, NY), MDA-MB-231 cells were cultured in Leibovitz’s L-15 medium (GIBCO, Grand Island, NY, USA). Both RPMI 1640 and L15 medium were supplemented with 10% fetal calf serum (Gibco by Life Technologies, Cergy Pontoise, France), and penicillin (100 U/mL) and streptomycin (100 μg/mL). For MCF-7 cells, the medium additionally contained human-recombinant insulin (10 μg/mL) to maintain the endocrine dependency. For MCF-7/ADR cells, the medium additionally contained 1 μg/mL adriamycin to maintain the drug resistance property. The cells were cultured at 37 °C in a humidified atmosphere under 5% CO_2_. The cells were subcultured every 2–4 days and harvested in the logarithmic phase of growth.

### Reagents and antibodies

Tamoxifen (4-hydroxytamoxifen, 4-OHT/TAM) and 5-aza (5-aza-2′-deoxycytidine, 5-aza-dC) were purchased from Sigma-Aldrich (St Louis, Missouri, USA). Indicated cells were treated with 5 μmol/L TAM for 48 h. E-cadherin, vimentin, ER-α, PR, and Her-2 antibodies were purchased from Cell Signaling Technology (Beverly, MA, USA). Actin, p-gp, DNMT1 and DNMT3a antibodies were purchased from Santa Cruz Biotechnology (USA).

### MTT assay

The effects of different agents on cell proliferation were measured using the MTT assay [14]. Briefly, indicated cells were seeded at 3 × 10^4^ per well in 96-well plates in quadruplicate and incubated overnight. Different concentrations of the test agents were then added and incubated for 5 days. Thereafter, 25 μL of MTT solution (5 mg/mL) was added to each well and the cells were incubated for another 4 h at 37 °C. After the incubation, the supernatants were removed carefully, and 200 μL of DMSO was added to each well. The cells were then lightly shaked for 10 min. Absorbance was measured at 570 nm in a Microplate Reader (Bio-Rad, CA, USA). Analysis of the obtained results was done using GraphPad Prism 5 computer program to evaluate cell proliferation rate and cytostatic rate. Untreated cells were used as controls.

### Transwell migration assay

Cells were washed in serum-free medium twice. The chemoinvasion assay was conducted using the 24-well chemotaxis chambers with 8 μm pores (Corning, NY, USA) according to the manufacturer’s instruction. Briefly, indicated cells were pretreated with or without TAM (5 μmol/L) for 48 h. Then, 2 × 10^4^cells were resuspended in fresh serum-free media and seeded into the upper chamber of a 24-well plate, while the lower chamber contained fresh culture media with 10% FBS as a chemo-attractant. The cells were allowed to invade for 24 h at 37 °C and the chambers were then washed with PBS. The cells on lower surface of the chamber were stained with 0.1% Giemsa stain solution for 2 h and counted in four different random fields at ×10 magnifications under electron microscope. Each experiment was performed at least three times.

### Western blot assay

Cells were washed twice with ice-cold PBS and solubilized in 1% Triton lysis buffer (50 mmol/L Tris-HCl, pH 7.4, 10 mmol/L EDTA, 100 mmol/L NaF, 150 mmol/L NaCl, 1% Triton X-100, 1 mmol/L PMSF 1 mmol/L Na_3_VO_4_, and 2 μg/mL aprotinin) on ice, then sonication and incubation at 4 °C for 30 min, followed by centrifugation at 12,000 g at 4 °C for 20 min. Then proteins were quantified according to BCA (Beyotime, China) method. Total proteins were subjected to SDS-polyacrylamide gel electrophoresis (SDS-PAGE) and electronically transferred to nitrocellulose membranes (Immobilon-P, Millipore, Bredford, MA, USA). After blocking with 5% skim milk in TBST (10 mmol/L Tris, pH 7.4, 150 mmol/L NaCl and 0.1% Tween-20) for 1 h, the bands were incubated in the indicated primary antibodies at 4 °C overnight, followed by secondary antibodies incubated for 30 min at room temperature. After washing with TBST, the proteins were detected using an enhanced chemiluminescence reagent (SuperSignal Western Pico Chemiluminescent Substrate, Pierce, USA) and visualized with an ECL detection system (DNR Bio-Imaging Systems, Jerusalem, Israel) [15].

### Immunofluorescence

The cells were seeded in Lab-Tek chamber slides (Nunc S/A, Polylabo, Strasbourg, France). The cells were treated with or without TAM (5 μmol/L) for 72 h and fixed with 4% paraformaldehyde for 15 min, permeabilized with 0.2% Triton X-100 for 5 min, blocked with 5% bovine serum albumin (BSA) in 1× PBS for 1 h at room temperature and then incubated with E-cadherin and vimentin antibodies for 1 h. Then Alexa Fluor 546-conjugated goat anti-rabbit IgG or Alexa Fluor 488-conjugated goat anti-rabbit IgG (Molecular Probes) were added in blocking solution for 1 h at room temperature in the dark. 4′6-diamidino-2- phenylindole was used to stain nuclei for 5 min. After mounted with the Slow Fade Antifade Kit (Molecular Probes, Eugene, OR, USA), the cells were visualized by fluorescence microscopy (BX61, Olympus, Japan) [16].

### RNA extraction and quantitative real-time PCR (qRT-PCR)

The cells were cultured and harvested at the indicated times. Total RNA was extracted from cells using the RNeasy mini kit (Qiagen, Carlsbad, CA, USA). For miRNAs, The One Step PrimeScript^®^ miRNA cDNA Synthesis Kit (Takara, Japan) was used for RNA reverse transcription. Relative expression of miRNAs was calculated via the comparative cycle threshold (Ct) method, and the expression of small nuclear RNA U6 was used as reference. The sequence-specific forward primers for mature miR-200c was: 5′-ACACTCCAGCTGGGTAATACTGCCGGGTAA-3′ and for U6 internal control was forward (5′-GCTTCGGCAGCACATATACTAAAAT-3′) and reverse (5′-CGCTTCACGAATTTGCGTGTCAT-3′), respectively. The Uni-miR qPCR Primer was included in the kit. SYBR^®^ Premix Ex Taq™ II (Perfect Real Time) (Takara, Japan) was used for monitoring the amount of miRNA. The PCR conditions were 30s at 95 °C, followed by 45 cycles at 95 °C for 5 s and 58 °C for 25 s. The relative amount of the target RNA was calculating by 2^-ΔΔCt^ method. The detailed method was described in our previous studies [16].

### MicroRNA microarray analysis

MCF-7 and MCF-7/ADR cells were cultured without insulin or adriamycin for 3 days. The expression levels of miRNAs were quantified using GeneChip miRNA Array (Affymetrix, Santa Clara, CA, USA) according to the manufacturer’s instructions by Gene Tech Biotechnology Company (Shanghai, China) [16]. In brief, total RNA (1 μg) was extracted with miRNeasy Mini Kit (Qiagen, Germany) and labeled with a FlashTag Biotin RNA Labeling kit (Genisphere, Hatfield, PA, USA). Then the labeled RNA was injected onto the microarrays and incubated at 48 °C for 16 h. After washing and staining, the signals were obtained using a GeneChip Scanner 3000 7G (Affymetrix, Santa Clara, CA). Data was normalized using the RMA algorithm. The PCA and unsupervised clustering of microarray analysis were shown in Additional files 1 and 2. QC report and raw data of microarray analysis were shown in Additional files 3 and 4. The microarray result was uploaded to GEO database. Data from this microarray is available at GSE96821.

### Transfection

The si-DNMT1 and si-DNMT3a and corresponding negative control were designed and synthesized by RiboBio (Guangzhou, China) and stored at −80 °C before use. Cells were transiently transfected with si-DNMT1 and si-DNMT3a and corresponding negative control using Lipofectamine 2000 reagent (Invitrogen) according to the manufacturer’s protocol.

### Xenograft study in nude mice

5-week-old female Balb/c nude mice were from SLAC Laboratory Animal Co., Ltd. (Shanghai, China). Mice were randomly allocated to two groups (*n* = 6 per group). 1 × 10^6^ MDA-MB-231 cells were injected into the tail vein of the control group. 1 × 10^6^ MDA-MB-231 T cells (MDA-MB-231 cells pretreated with 5 μmol/l TAM for 5 d) were injected into the tail vein of the treatment group and given with TAM orally every other day for 2 months. Mice were killed by cervical dislocation according to the protocol filed with the Guidance of Institutional Animal Care and Use Committee of China Medical University, and lung tissue was taken out for HE staining to ensure metastasis focals. Experimental research on mice complied with the Guidance of Institutional Animal Care and Use Committee of China Medical University, and had been approved by the ethics committee of China Medical University.

### CpG island predictor

The CpG island was predicted by the MethPrimer software. MethPrimer accepts a DNA sequence as input, performs a digital bisulfite conversion of the input sequence, and then picks primers on the converted sequence. Results of primer selection are delivered through a Web browser in text and graphic views (http://www.urogene.org/cgi-bin/methprimer/methprimer.cgi) [17]. The miR-200c promoter region used an island size of 3000 nucleotides, a GC percentage of at least 50% and an observation/expectation CpG ratio of more than 0.6.

### Statistical analysis

All the presented data were expressed as the mean ± SD and the representative results were confirmed in at least three independent experiments. Statistical comparisons were calculated by Student’s *t*-test. *P* < 0.05 was considered statistically significant. IC_50_ values were calculated by nonlinear regression analysis using GraphPad Prism 5 software.

## Results

### Differences in EMT character of breast cancer cell lines

**Fig. 1:**
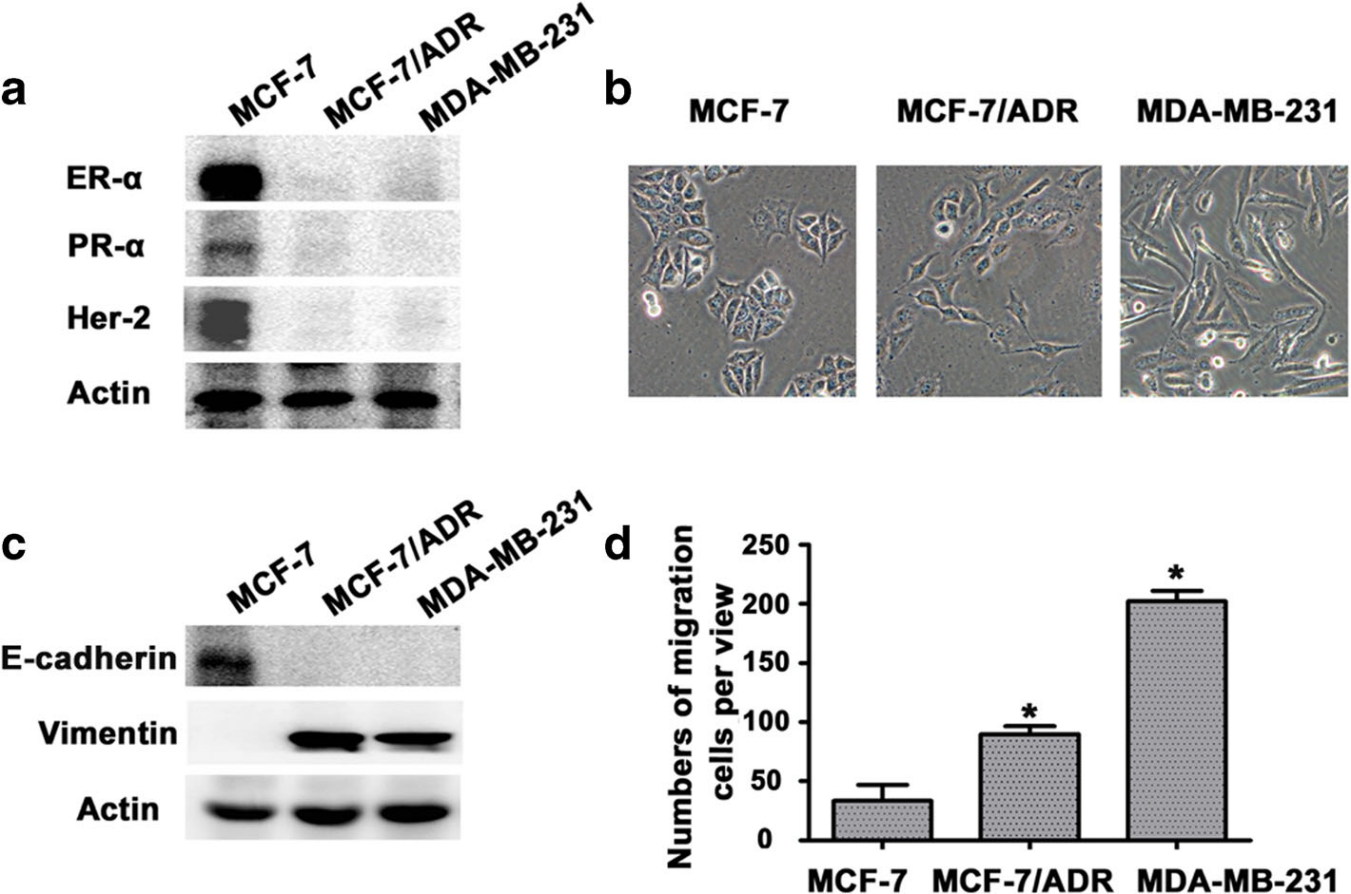
Differences in EMT-like character of breast cancer cell lines. **a**, Western blot analysis showed the expression of ER-α, PR-α and Her-2 in the indicated cells. **b**, Morphology showed by electron microscope at high magnification(×40) in the indicated cells. **c**, Western blot analysis showed the expression of E-cadherin and Vimentin, in the indicated cells. **d**, Migration ability of indicated cells were subjected to migration assay, the numbers of migration cells were represented by the mean of three individual experiments. Data were expressed as mean ± SD [*n* = 3, statistical significance relative to control (con)]. * *P* < 0.05

Usually, TNBC cells are divided into two types: cells that were initially TNBC and those that transformed from HR^+^ breast cancer cells [18, 19]. Here we used both kinds of TNBC cells to fully illustrate the character of TNBC [20, 21], with HR^+^ breast cancer cell MCF-7 taken as control. To confirm the character of breast cancer cells that we use, the Her2, PR, ER-α expression were determined in three different breast cancer cell lines. MCF-7 cells are ER^+^/PR^+^/Her2^−^ with epithelial morphology (Fig. 1a, b); MCF-7/ADR and MDA-MB-231 cells are TNBC (ER^−^/PR^−^/Her2^−^) with mesenchymal morphology (Fig. 1b). We also examined the EMT markers, E-cadherin and vimentin (Fig. 1c). We then examined the migration ability of the three breast cancer cell lines. The mesenchymal TNBC cells were more likely to metastasize than the epithelial TNBC cells and non-TNBC cells, (*P* < 0.05; Fig. 1d), which was facilitated by the EMT process.

### TAM reversed EMT in mesenchymal TNBC cells

**Fig. 2:**
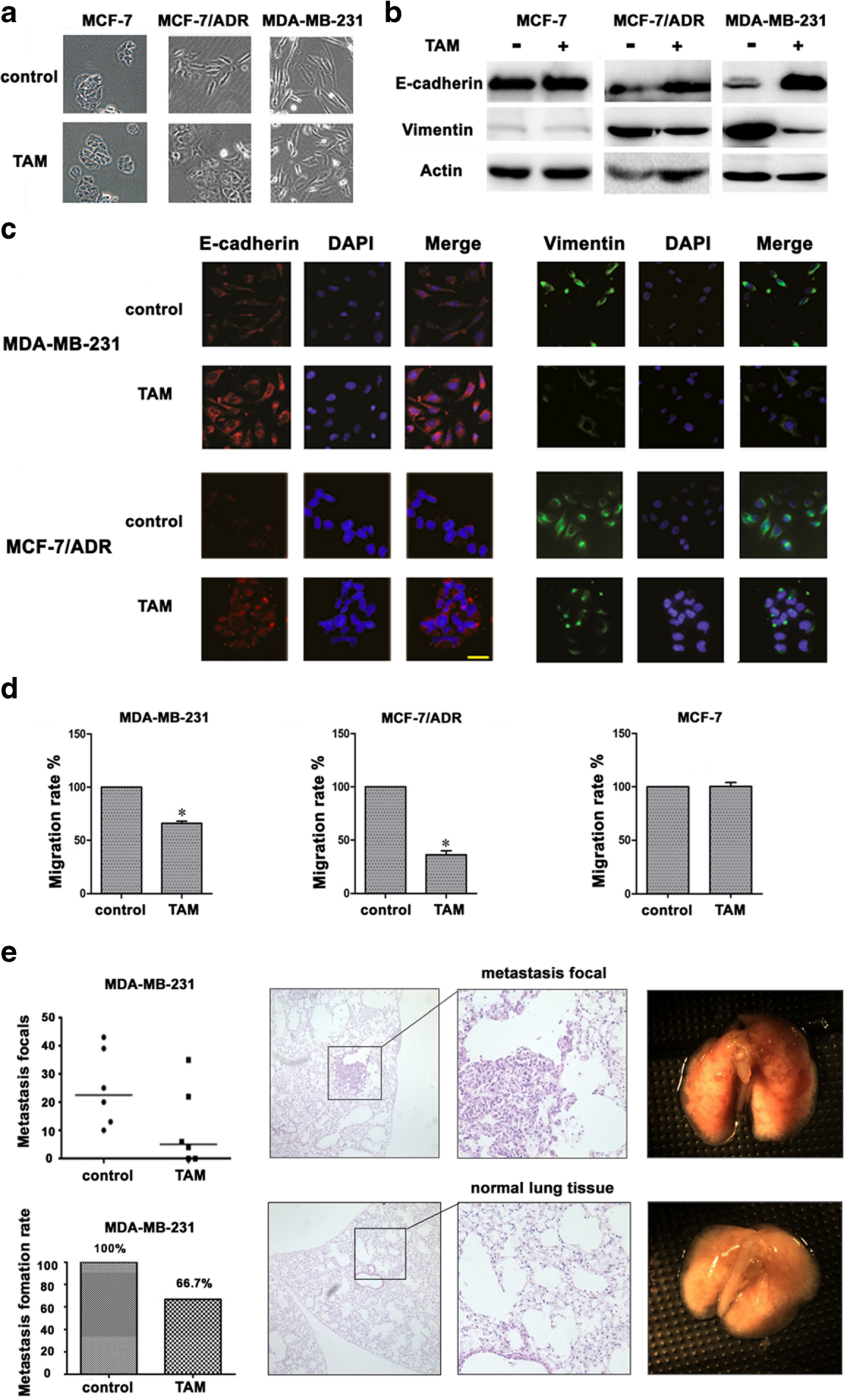
TAM reversed EMT in mesenchymal TNBC cells. **a**, Morphology showed by electron microscope at high magnification(×40) in the indicated cells treated with or without TAM (5 μmol/L for 48 h). **b**, Western blot analysis showed the expression of E-cadherin and Vimentin in the indicated cells treated with or without TAM (5 μmol/L for 48 h). **c**, Immunofluorescence analysis showed the expression of E-cadherin (*red*), Vimentin (*green*) in the indicated cells treated with or without TAM (5 μmol/L for 48 h). Image were photographed by electron microscope at high magnification(×40). **d**, Migration ability of indicated cells treated with or without TAM were subjected to migration assay, the numbers of migration cells were represented by mean of three individual experiments. **e**, Migration ability of indicated cells treated with or without TAM were subjected to lung metastasis focals and metastasis rate; HE staining of lung tissue from mice were photographed by electron microscope at high magnification(×40). up, lung metastasis focal of breast cancer; down, normal lung sample without malignancy

MCF-7, MCF-7/ADR and MDA-MB-231 breast cancer cell lines were cultured overnight and treated with TAM (0 [control], 1, or 5 μmol/L) for 48 h. Dramatic morphological changes were observed in mesenchymal TNBC cells MCF-7/ADR and MDA-MB-231 at 5 μmol/L (Fig. 2a), which transformed from characteristic spindle-shaped mesenchymal cells to epithelial cells with tight junctions. However, this morphological change was less obvious in cells treated with 1 μmol/L TAM. The MCF-7 cells retained their epithelial character after treated with TAM (Additional file 5). Therefore, we selected 5 μmol/L TAM as our treatment concentration (Fig. 2a).

These results were also confirmed by detection of EMT protein markers and immunofluorescence tests. Protein samples from MDA-MB-231 and MCF-7/ADR cells treated with TAM showed increased E-cadherin and decreased vimentin compared to control group (Fig. 2b, c). TAM also dramatically decreased the cell migration ability to 59.4 ± 3.8% (MDA-MB-231) and 43.2 ± 5.7% (MCF-7/ADR cells) compared to that of the control group (Fig. 2d).

We also used a murine model to test the effect of TAM on migration ability of mesenchymal TNBC cells. MDA-MB-231 cells were pretreated with TAM for 5 days, and then injected into tail vein of mice, which also received TAM by gavage every other day. The mice were sacrificed at 8 weeks, and their lungs were removed, sectioned into pathological samples, and stained with hematoxylin-eosin (HE) staining to visualize metastases (Fig. 2e). The rate of migration to lungs of treated mice was 66.7%, compared with 100% of the control group (Fig. 2e). These data thus imply that TAM decreased migration ability by reversing EMT process of TNBC cells.

### TAM increased chemosensitivity in TNBC cells

**Fig. 3:**
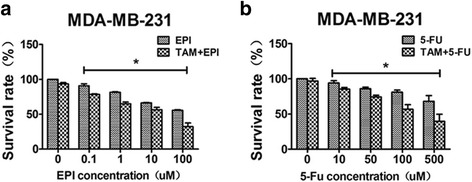
TAM increased the chemosensitivity in TNBC cells. **a**, **b**, Effect of epirubicin and 5-Fu on survival rate in MDA-MB-231 cells pretreated with or without TAM (5 μmol/L). The survival rate of cells in vehicle was taken as 100%. All experiments were repeated 3 times. Results were expressed as mean ± SD (*n* = 3). * *P* < 0.05

Given that EMT affects chemosensitivity of breast cancer [17], we hypothesized that reversing EMT in mesenchymal TNBC cells might increase their sensitivity to chemotherapeutic agents. We evaluated the sensitivity of commonly used drugs, epirubicin and 5-Fluorouracil (5-Fu), in breast cancer cells by MTT assay, and found that after 5 days’ pre-treatment with TAM, sensitivity to epirubicin increased significantly in MDA-MB-231 cells (Fig. 3a), with the IC_50_ of 12.05 μmol/l, whereas the IC_50_ of the control group could not be reached due to chemoresistance. Similarly, sensitivity to 5-Fu increased in MDA-MB-231 cells (IC_50_: 220.4 μmol/l; Fig. 3b). These results thus indicate that TAM increased the sensitivity of mesenchymal TNBC cells.

### TAM reversed EMT by up-regulating miR-200c

**Fig. 4:**
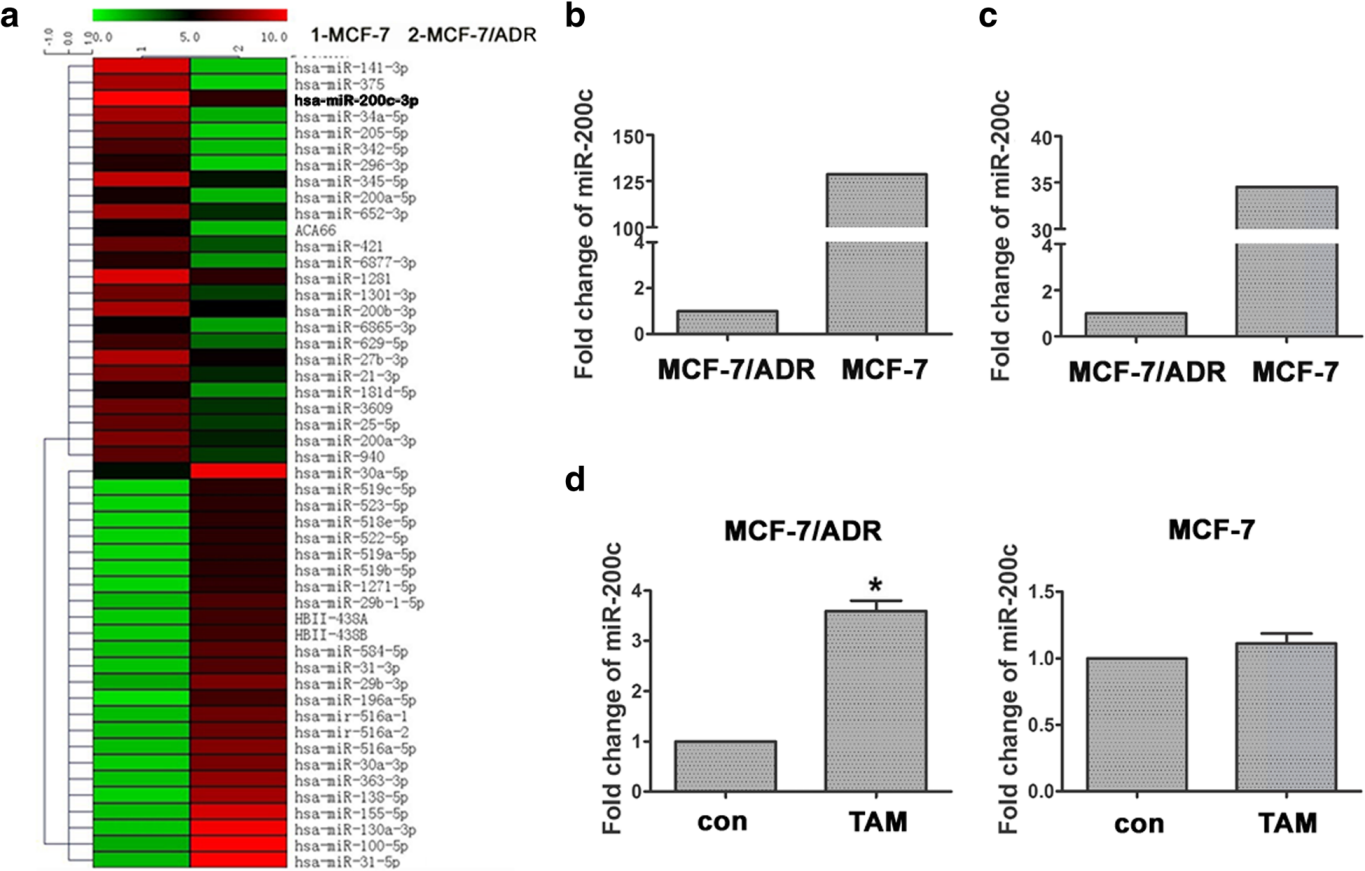
TAM reversed EMT by up-regulating miR-200c of mesenchymal TNBC cells. **a**, Comparison of miRNAs expression in MCF-7/ADR cells and MCF-7 cells was performed by microarray analysis. After normalization, differentially expressed (>2 fold) miRNAs were identified through Volcano Plot filtering. **b**, Relative expression of miR-200c in MCF-7 and MCF-7/ADR cells determined by microarray analysis. **c**, Relative expression of miR-200c in MCF-7 and MCF-7/ADR cells determined by realtime-PCR. **d**, Relative expression of miR-200c cells in MCF-7 and MCF-7/ADR cells treated with or without TAM for 48 h determined by realtime-PCR. miRNA expression were normalized to small nuclear ribonucleic acid (snRNA) RNU6B, and results were expressed as fold change relative to control (con). All experiments were repeated 3 times. Results were expressed as mean ± SD (*n* = 3). * *P* < 0.05

To determine the effect of TAM on miRNAs that mediate regulation of EMT in TNBC cells, we compared miRNA microarray analysis of TNBC cells (MCF-7/ADR) with non-TNBC cells (MCF-7). After normalization, we found significantly different miRNA expression between the two cell lines, using Volcano Plot filtering of 400 miRNAs (Fig. 4a). Consistent with a recent report [22], miR-200c expression, which is commonly acknowledged to affect EMT, showed dramatic difference between the two cell lines. Relative fold change was 128.6-fold in MCF-7 cells in the expression of miR-200c compared with MCF-7/ADR cells (Fig. 4b). To verify the result of microarray analysis, we performed quantitative real-time PCR for the two cell lines. Consistent with the microarray analysis result, the relative fold change was 34.508-fold in MCF-7 cells in the expression of miR-200c compared with MCF-7/ADR cells (Fig. 4c). However, after treatment with 5 μmol/L TAM for 48 h, miR-200c expression restored in the mesenchymal MCF-7ADR cells; this treatment did not affect miR-200c expression in non-TNBC cells MCF-7 (Fig. 4d). These results suggested that TAM could induce increased miR-200c in mesenchymal TNBC cells.

### miR-200c promoter regions were hypermethylated in TNBC cells

**Fig. 5:**
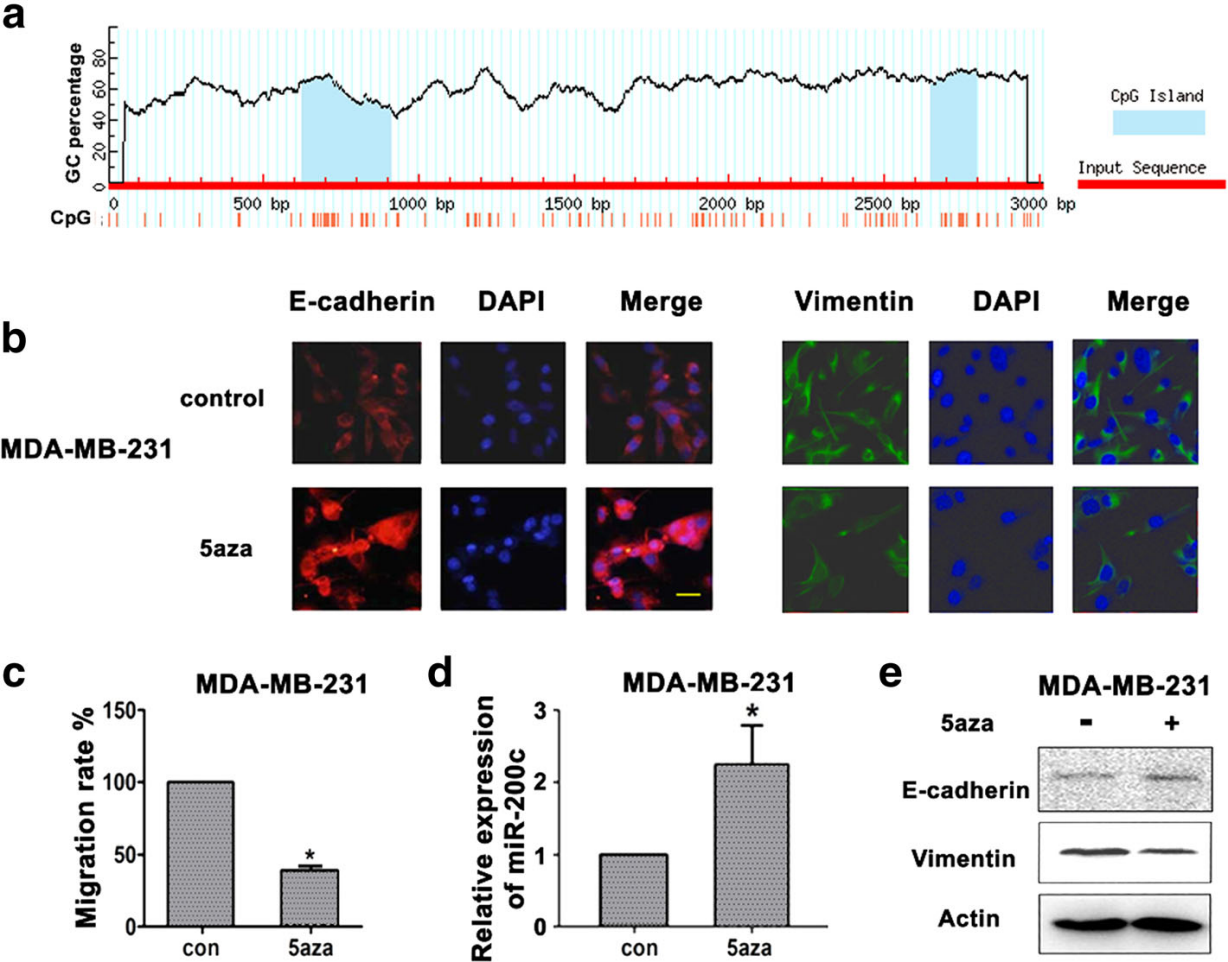
Promoter region of miR-200c were hypermethylated in TNBC cells. **a**, An online CpG island predictor was used to detect the CpG islands of 3000 bp of miR-200c promoter sequence, upstream of the miR-200c cluster. **b**, Immunofluorescence analysis showed the expression of E-cadherin (*red*), Vimentin (*green*) in MDA-MB-231 cells treated with or without 5aza for 72 h. Image were photographed by electron microscope at high magnification(×40). **c**, Migration ability of MDA-MB-231 cells were subjected to migration assay, the numbers of migration cells were represented by the mean of three individual experiments. Data were expressed as mean ± SD (*n* = 3), * *P* < 0.05; **d**, Relative expressions of miR-200c cells in MDA-MB-231 cells treated with or without 5aza for 72 h determined by realtime -PCR. miRNA expressions were normalized to small nuclear ribonucleic acid (snRNA) RNU6B, and results were expressed as fold change relative to control (con). **e**, Western blot analysis showed the expression of E-cadherin and Vimentin in MDA-MB-231 cells treated with or without 5aza for 72 h. All experiments were repeated 3 times. Results were expressed as mean ± SD (*n* = 3). * *P* < 0.05

We hypothesized that hypermethylation of the miR-200c promoter region might have caused low miR-200c expression in mesenchymal TNBC cells. This mechanism of miRNA down-regulation is closely related to DNA hypermethylation in breast cancer [23]. As miRNA expression is affected by methylation of CpG islands in their promoter regions, we used an online CpG island predictor to confirm the hypothesis [24]. Sequence analysis of miR-200c promoters revealed the presence of a highly-expressed CpG island upstream of the miR-200c cluster (blue, Fig. 5a). This prediction indicated that miR-200c expression might be affected by demethylation of its promoters. To evaluate the demethylation effect on miR-200c expression, we examined miR-200c expression after treatment with 5-azacytidine (5-AZA), a specific inhibitor of DNA methylation, for 72 h. Quantitative real-time PCR revealed that the relative fold change of miR-200c was 2.23 ± 0.67 -fold after treatment with 5-AZA for 3 days, compared with negative control, using MDA-MB-231 cells (*P* < 0.05; Fig. 5d). Then EMT phenotypic change was detected by immunofluorescence. Expression of E-cadherin increased and vimentin decreased in MDA-MB-231 cells (Fig. 5b) accompanied with decreased migration ability. Transwell assay also showed that after pretreatment of 5-AZA, cell migration ability decreased to 39 ± 3.57% compared to that of control, in MDA-MB-231 cells. (Figure 5c). These results suggested that 5-AZA also reversed EMT via increased miR-200c expression, and decreased migration ability in TNBC cells.

### TAM reversed EMT by inhibiting the expression of DNMT1/DNMT3a

**Fig. 6:**
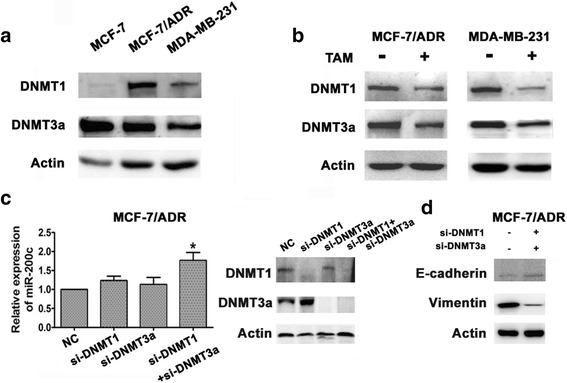
TAM reversed EMT by inhibiting the expression of DNMT1/DNMT3a. **a**, Western blot analysis showed the expression of DNMT1 and DNMT3a in indicated cells. **b**, Western blot analysis showed the expression of DNMT1 and DNMT3a in MCF-7/ADR and MDA-MB-231 cells treated with or without TAM for 48 h. **c**, MCF-7/ADR cells were transfected with either NC or si-DNMT1 or si-DNMT3a or si-DNMT1 and si-DNMT3a for 48 h; Relative expressions of miR-200c were determined by realtime-PCR in indicated cells. **d**, Western blot analysis showed the expression of E-cadherin and Vimentin in double knocked (si-DNMT1 and si-DNMT3a) MCF-7/ADR cells. All experiments were repeated 3 times. Results are expressed as mean ± SD (*n* = 3). * *P* < 0.05

Our data demonstrated that TAM could change the morphology of mesenchymal TNBC cells, and reverse the EMT process by regulating the expression of miR-200c. A previous study showed that miRNA expression was tightly controlled by DNA methylation [25]. Therefore, we use western blot method to determine the expression of DNA-methyltransferase (DNMT, an essential enzyme in the process of DNA methylation), to evaluate DNA methylation levels. Methylation status differs among breast cancer cell lines; mesenchymal TNBC cells showed higher methylation status than MCF-7 cells (Fig. 6a). We discovered that the DNMT expression in mesenchymal TNBC cells decreased after 48 h’ treatment with TAM (Fig. 6b). Western blot revealed significant decreased DNMT1 and DNMT3a in MDA-MB-231 and MCF-7/ADR cells. To confirm that miR-200c is regulated by DNMTs, we used siRNAs to double-knock *DNMT1* and *DNMT3a*. Compared with scrambled siRNA control cells, knockdown of *DNMT1* and *DNMT3a* significantly increased the miR-200c expression (Fig. 6c), accompanying with decreased vimentin and increased E-cadherin in MDA-MB-231 and MCF-7/ADR cells (Fig. 6d). Together, these data suggested that TAM increased miR-200c by partially inhibition of DNMT1 and DNMT3a to reverse EMT in mesenchymal TNBC cells.

## Discussion

TNBC is an aggressive breast cancer subtype that metastasizes early and is associated with poor overall survival. Preventing metastasis is hampered by limited treatment options. TAM is an ER antagonist, mainly used in ER^+^ breast cancers through ER-dependent mechanism with definitive efficacy [9–11, 19, 26–31], and is thus much less efficacious in ER^−^ breast cancers, such as TNBC [32]. Recently, TAM showed broad-spectrum antitumor properties in ER^−^ cancers, such as gastric cancer, colorectal cancer and cholangiocarcinoma [33]. In gastric cancer cells, TAM could inhibit the PI3K/Akt signaling pathway [7]. TAM could also decreased P-gp expression, to reverse multidrug resistance in QBC939/ADM cholangiocarcinoma cells [9]. However, in our results, P-gp expression barely changed in TNBC cells after TAM treatment, which implied that their increased chemosensitivity occurred through EMT changes.

Characteristics of TNBC cells are similar to cells that undergo EMT. Their migratory and invasive properties increase while their adhesive properties decrease as epithelial cells transform to mesenchymal cells. The relationship between TAM and EMT was reported in some TAM-resistant breast cancers: after acquiring endocrine resistance, most of breast cancer cells grew as loosely packed colonies, underwent EMT morphological changes and altered their growth rate and increased aggressive behavior [34]. However, the relationship between TAM and EMT is not restricted to breast cancer. Similar researches were reported in ER^−^ endometrial cancer. After long exposure to TAM, epithelial-like endometrial cancer cells underwent EMT, and their ER status changed from ER^+^ to ER^−^ [12]. This research demonstrated that long exposure to TAM by epithelial cells might induce phenotypical changes, through a mechanism unrelated to ER status. However, the process of EMT was reversible (mesenchymal–epithelial transition; MET), although no reports are available on whether TAM could reverse EMT in mesenchymal breast cancer cells. Our study showed that TAM could change the mesenchymal phenotype of breast cancer cells by reversing EMT. EMT is closely associated with metastasis, drug resistance and progression of malignant tumors [35–38]. Our study provides the first evidence to suggest that TAM could reverse EMT and decrease migratory ability in vitro and vivo, while concomitantly increasing chemosensitivity in mesenchymal TNBC cells.

To explore the mechanism of MET induced by TAM on mesenchymal TNBC cells, we compared miRNA expression between mesenchymal and epithelial breast cancer cells, using microarray analysis. Among the most significant miRNAs, we focused on the regulation of miRNAs that affect the EMT process. The reported role of the miR-200 family as EMT inhibitors, to reduce tumor cell migration was confirmed by our microarray analysis [39]. MiR-200c could promote breast cancer cell epithelial identity, and repress related genes that regulate E-cadherin and cell polarity [40], and reportedly regulates the EMT process directly, thus affecting chemosensitivity [17]. Reciprocally, the process of EMT could also regulate miR-200c expression [17]. Therefore, the study we report here is based on the known ability of miRNA-200s family to reverse the process of EMT.

However, to our knowledge, this is the first report of the mechanism of how TAM regulates miR-200c. MiRNAs are highly conserved sequences with promoters of about 2000 base pairs [41]. As DNA methylation can change promoter activity [42], we used online software to predict the methylation status of miR-200c promoters. We also verified the existence of CpG islands in miR-200c promoters, and high expression of DNMTs in mesenchymal TNBC cells. Expression and methylation status of miR-200 might be useful as markers for EMT in breast cancer [43]. The result was consistence with the research of Vrba, et al. who also showed the important role of DNA methylation in regulating miR-200c expression and the control of phenotypic conversions in cancer cells [44]. After TAM treatment, DNMT expression decreased and miR-200c expression increased in TNBC cells. These results imply that TAM regulate miR-200c expression by downregulating DNMT expression. As similar results were seen with the demethylation agent 5-AZA, we considered that 5-AZA could also downregulate DNMT expression and reactivate the gene function turned off by hypermethylation [45, 46]. To test our hypothesis, we knocked down *DNMT1* and *DNMT3a* using siRNAs. Our result showed that double knockdown of *DNMT1* and *DNMT3a* also upregulated miR-200c, which confirmed the relationship between miRNA and hypermethylation. Therefore, TAM can apparently reverse EMT by downregulating DNMTs, thus increasing miR-200c expression.

## Conclusions

In conclusion, we verified that TAM up-regulates miR-200c expression in mesenchymal TNBC cell lines MDA-MB-231 and MCF-7/ADR, by downregulating DNMT expression, thus attenuating cell migratory capacities in vivo and in vitro. The MET process increased chemosensitivity of mesenchymal TNBC cells in vivo and vitro. The present study expands the effect of TAM, and may explain why some ER^−^ breast cancers respond to TAM. Our results suggest that TAM could be a DNMT inhibitor, thus indicating a wider range of both research in, and clinical use for, TAM in TNBC.

## Additional files

Additional file 1: PCA of microarray analysis. 1 represented for MCF-7, and 2 represented for MCF-7/ADR. (PNG 146 kb)

Additional file 2: Unsupervised clustering of microarray analysis. 1 represented for MCF-7, and 2 represented for MCF-7/ADR. (PDF 436 kb)

Additional file 3: QC report of microarray analysis. 1 represented for MCF-7, and 2 represented for MCF-7/ADR. (TXT 4 kb)

Additional file 4: Raw data of microarray analysis. Full list of deregulated miRNAs (>2 fold). 1 represented for MCF-7, and 2 represented for MCF-7/ADR. (TXT 50 kb)

Additional file 5: Morphology changes at different concentrations with TAM. Morphology of three breast cancer cell lines with or without treatment of TAM (0, 1 and 5 μmol/L for 48 h) were photographed by electron microscope at high magnification (×40). (TIFF 4122 kb)

## Abbreviations

EMT: Epithelial-to-mesenchymal transition
ER: Estrogen receptor
HR: Hormonal receptor
MAPK: Mitogen-activated protein kinase
PKC: Protein kinase C
TAM: Tamoxifen
TGF-β: Transforming growth factor-β
TNBC: Triple-negative breast cancer
