# Supplementary figures and images for: Tamoxifen reverses epithelial–mesenchymal transition by demethylating miR-200c in triple-negative breast cancer cells
Source: BMC Cancer. 2017 Jul 19;17:492. doi: 10.1186/s12885-017-3457-4 (PMC5518097; doi:10.1186/s12885-017-3457-4)

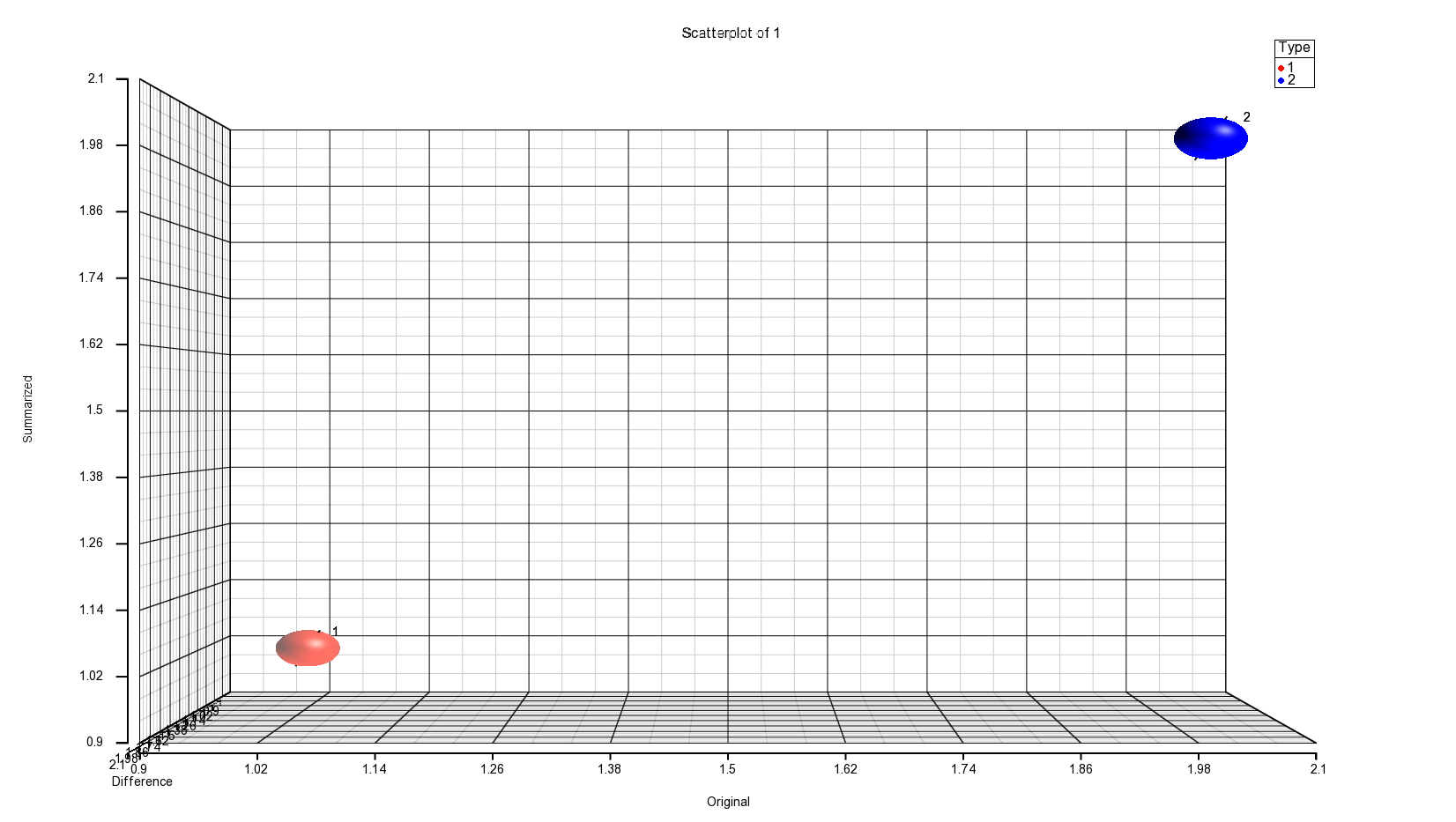

Supplement: Supplementary file 1 — PCA of microarray analysis. 1 represented for MCF-7, and 2 represented for MCF-7/ADR. (PNG 146 kb) [file 12885_2017_3457_MOESM1_ESM.png]

1

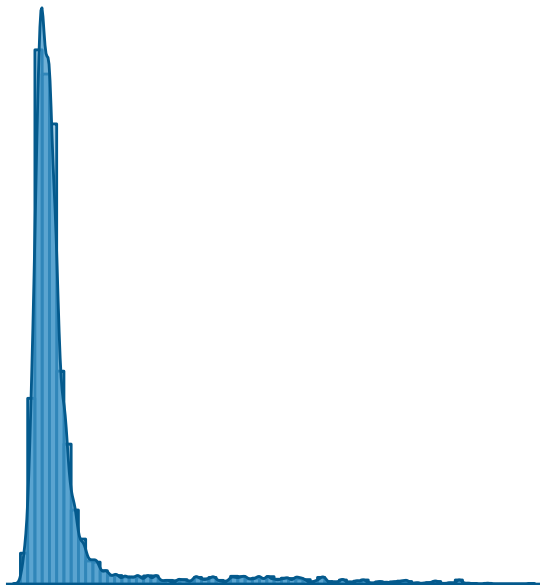

0.913

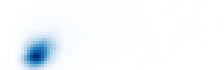

2

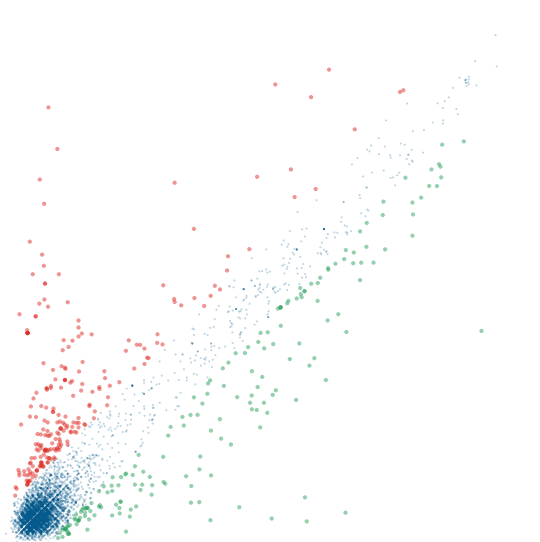

1

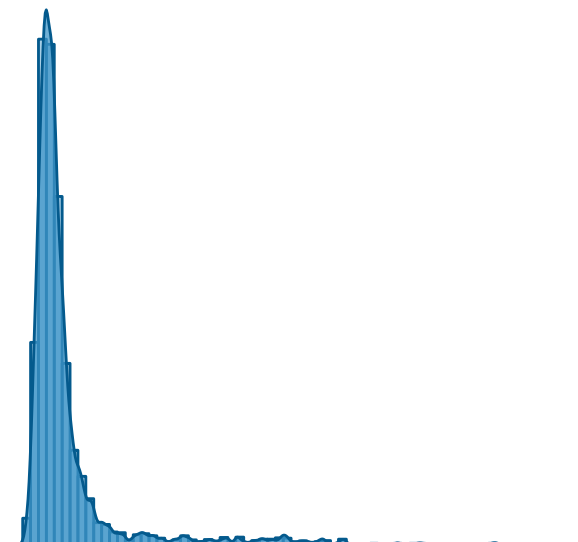

2

Supplement: Supplementary file 2 — Unsupervised clustering of microarray analysis. 1 represented for MCF-7, and 2 represented for MCF-7/ADR. (PDF 436 kb) [file 12885_2017_3457_MOESM2_ESM.pdf]

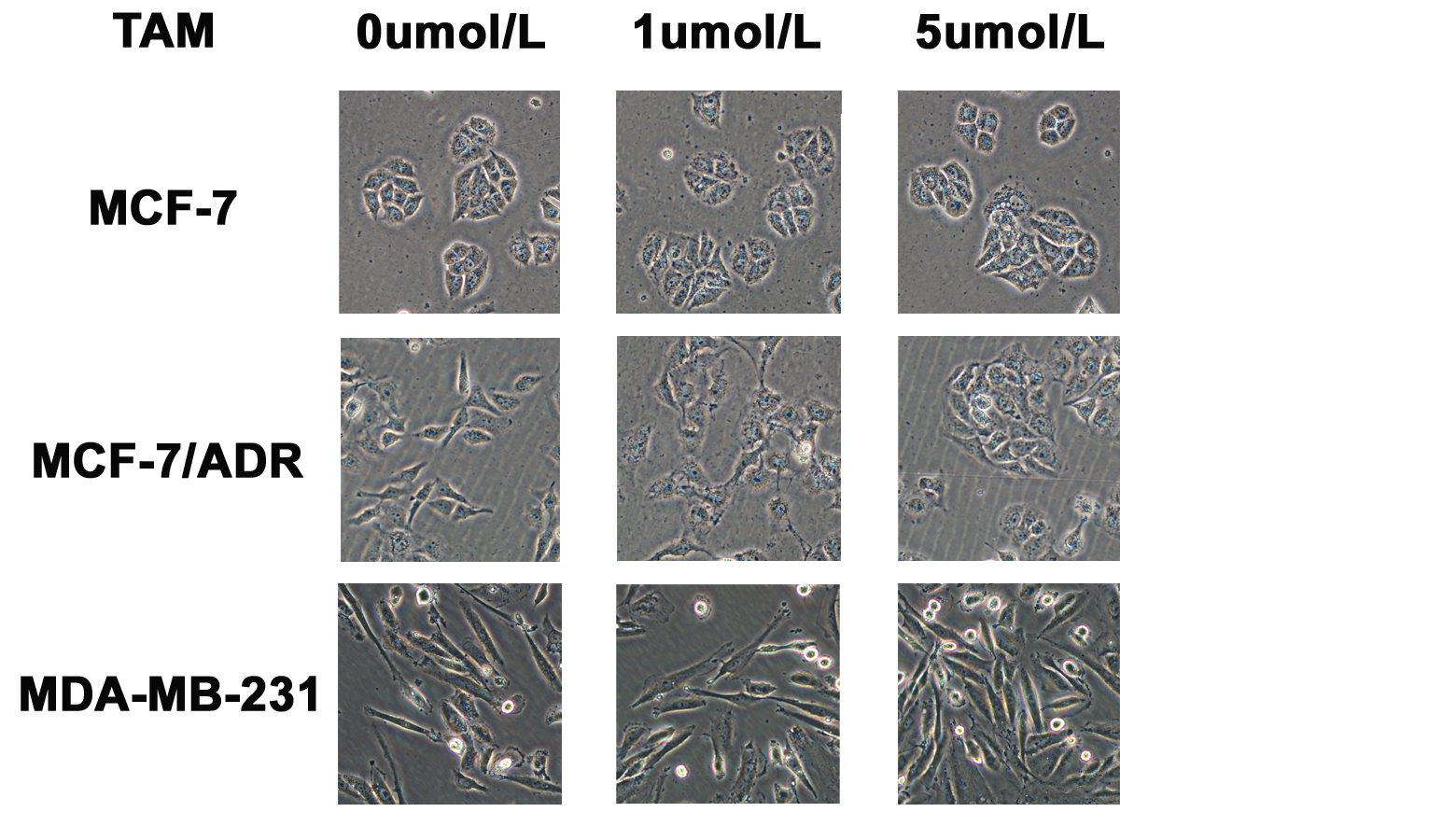

Supplement: Supplementary file 5 — Morphology changes at different concentrations with TAM. Morphology of three breast cancer cell lines with or without treatment of TAM (0, 1 and 5 μmol/L for 48 h) were photographed by electron microscope at high magnification (×40). (TIFF 4122 kb) [file 12885_2017_3457_MOESM5_ESM.tif]
